# Tooth Agenesis Patterns in Orofacial Clefting Using Tooth Agenesis Code: A Meta-Analysis

**DOI:** 10.3390/dj10070128

**Published:** 2022-07-05

**Authors:** Brian J. Howe, Chandler Pendleton, Miyuraj Harishchandra Hikkaduwa Withanage, Christopher A. Childs, Erliang Zeng, Arjen van Wijk, Ruurd Hermus, Carmencita Padilla, Jacqueline T. Hecht, Fernando A. Poletta, Iêda M. Orioli, Carmen J. Buxó-Martínez, Frederic Deleyiannis, Alexandre R. Vieira, Azeez Butali, Consuelo Valencia-Ramirez, Claudia Restrepo Muñeton, George L. Wehby, Seth M. Weinberg, Mary L. Marazita, Lina M. Moreno Uribe, Xian-Jin Xie

**Affiliations:** 1 Department of Family Dentistry, College of Dentistry, University of Iowa, Iowa City, IA 52242, USA; 2 The Iowa Institute for Oral Health Research, College of Dentistry, University of Iowa, Iowa City, IA 52242, USA; chandler-pendleton@uiowa.edu (C.P.); miyuraj-withanage@uiowa.edu (M.H.H.W.); erliang-zeng@uiowa.edu (E.Z.); azeez-butali@uiowa.edu (A.B.); lina-moreno@uiowa.edu (L.M.M.U.); xianjin-xie@uiowa.edu (X.-J.X.); 3 Hardin Library for Health Science, University of Iowa, Iowa City, IA 52242, USA; chris-childs@uiowa.edu; 4 Department of Biostatistics, College of Public Health, University of Iowa, Iowa City, IA 52242, USA; 5 Department of Social Dentistry and Behavioral Sciences, ACTA, 1012 WX Amsterdam, The Netherlands; a.v.wijk@acta.nl; 6 Orthopraktijk Capelle, 2904 EP Capelle aan den IJssel, The Netherlands; ruurdhermus@hotmail.com; 7 Department of Pediatrics, College of Medicine, University of the Philippines, Manila 1000, Philippines; cdpadilla1@up.edu.ph; 8 Department of Pediatrics, University of Texas Health Science Center at Houston, Houston, TX 77030, USA; jacqueline.t.hecht@uth.tmc.edu; 9 ECLAMC at Center for Medical Education and Clinical Research, CEMIC-CONICET, Buenos Aires 4102, Argentina; fpoletta@eclamc.org; 10 ECLAMC at Department of Genetics, Institute of Biology, Federal University of Rio de Janeiro, Rio de Janeiro 21941-901, Brazil; idea.orioli@gmail.com; 11 Dental and Craniofacial Genomics Core, School of Dental Medicine, University of Puerto Rico, San Juan 00925, Puerto Rico; carmen.buxo@upr.edu; 12 UCHealth Medical Group, Colorado Springs, CO 80920, USA; frederic.deleyiannis@uchealth.org; 13 Department of Oral and Craniofacial Sciences, School of Dental Medicine, University of Pittsburgh, Pittsburgh, PA 15260, USA; arv11@pitt.edu (A.R.V.); smwst46@pitt.edu (S.M.W.); marazita@pitt.edu (M.L.M.); 14 Department of Oral Pathology, Radiology, and Medicine, University of Iowa, Iowa City, IA 52242, USA; 15 Clinica Noel, Calle 14 No43B 146, Poblado Barrio Manila, Medellin 050034, Colombia; convalra@une.net.co (C.V.-R.); claudia.restrepo@clinicanoel.org.co (C.R.M.); 16 Department of Health Management and Policy, College of Public Health, University of Iowa, Iowa City, IA 52242, USA; george-wehby@uiowa.edu; 17 Department of Orthodontics, College of Dentistry, University of Iowa, Iowa City, IA 52242, USA

**Keywords:** cleft lip and palate, hypodontia, machine learning, phenotype

## Abstract

Individuals with orofacial clefting (OFC) have a higher prevalence of tooth agenesis (TA) overall. Neither the precise etiology of TA, nor whether TA occurs in patterns that differ by gender or cleft type is yet known. This meta-analysis aims to identify the spectrum of tooth agenesis patterns in subjects with non-syndromic OFC and controls using the Tooth Agenesis Code (TAC) program. An indexed search of databases (PubMed, EMBASE, and CINAHL) along with cross-referencing and hand searches were completed from May to June 2019 and re-run in February 2022. Additionally, unpublished TAC data from 914 individuals with OFC and 932 controls were included. TAC pattern frequencies per study were analyzed using a random effects meta-analysis model. A thorough review of 45 records retrieved resulted in 4 articles meeting eligibility criteria, comprising 2182 subjects with OFC and 3171 controls. No TA (0.0.0.0) was seen in 51% of OFC cases and 97% of controls. TAC patterns 0.2.0.0, 2.0.0.0, and 2.2.0.0 indicating uni- or bi-lateral missing upper laterals, and 16.0.0.0 indicating missing upper right second premolar, were more common in subjects with OFC. Subjects with OFC have unique TA patterns and defining these patterns will help increase our understanding of the complex etiology underlying TA.

## 1. Introduction

Children with oral clefts show a wide range of dental anomalies, expanding the phenotypic spectrum of non-syndromic orofacial clefting (OFC) beyond the primary defect. These anomalies affect the size, shape, number, symmetry, position, and eruption of teeth [1,2,3,4,5]. Tooth agenesis is a common dental anomaly in the general population, occurring in the permanent dentition at a rate of 0.6–5.2%. In subjects with orofacial clefting, however, tooth agenesis (TA) is one of the most common dental anomalies, with an estimated prevalence of 9.3–40.4% in the permanent dentition [1,5,6,7,8]. It can be a challenge to delineate the root cause of TA when a cleft is present. TA may share a common genetic etiology with OFC, as indicated by studies reporting more than 26 genes, including but not limited to: MSX1, PAX9, IRF6, TP63, BMP2, BMP4, WNT10A, WNT3, and AXIN2, associated with the co-occurrence of OFC [9,10]. Alternatively, TA may be the physical consequence of the cleft defect itself due to deficiencies in mesenchymal tissue or blood supply [11] or may be caused by surgical repair [12]. The overall pattern of TA can provide clues. However, few studies have examined patterns of tooth agenesis in individuals with OFC and how factors such as ethnicity, sex, cleft laterality, and cleft type impact those patterns [13,14,15,16].

To detect patterns in dental anomalies, the Tooth Agenesis Code (TAC) was developed by van Wijk and Tan as a binary coded algorithm with a unique value assigned to each tooth to identify simultaneously missing teeth due to agenesis [17]. TAC has been utilized in previous studies to identify patterns of tooth agenesis in subjects with OFC and varying results have been found [13,14,15,18,19]. TAC has also been used in the general population [16,20] as well as those with known syndromes such as Apert, Crouzon, and Pierre Robin [21,22,23,24]. To date, studies examining agenesis patterns in subjects with OFCs using TAC have not used controls and thus are unable to discern how TAC patterns differ from those in the general population. The aims of this study are to: (1) identify if discernable patterns of tooth agenesis occur in subjects with OFC compared with the general population using a meta-analysis methodology, (2) characterize patterns of tooth agenesis in the largest international cohort (OFC1) to date of children with non-syndromic orofacial clefting and controls, and (3) to test how gender, cleft type, and cleft laterality fit into the patterns identified.

This large sample will allow us to more conclusively test the hypothesis that subjects with OFC have unique patterns of tooth agenesis compared to the general population. The null hypothesis is that there will be no difference in the TAC patterns in subjects with OFC vs. controls.

## 2. Materials and Methods

**Figure 1:**
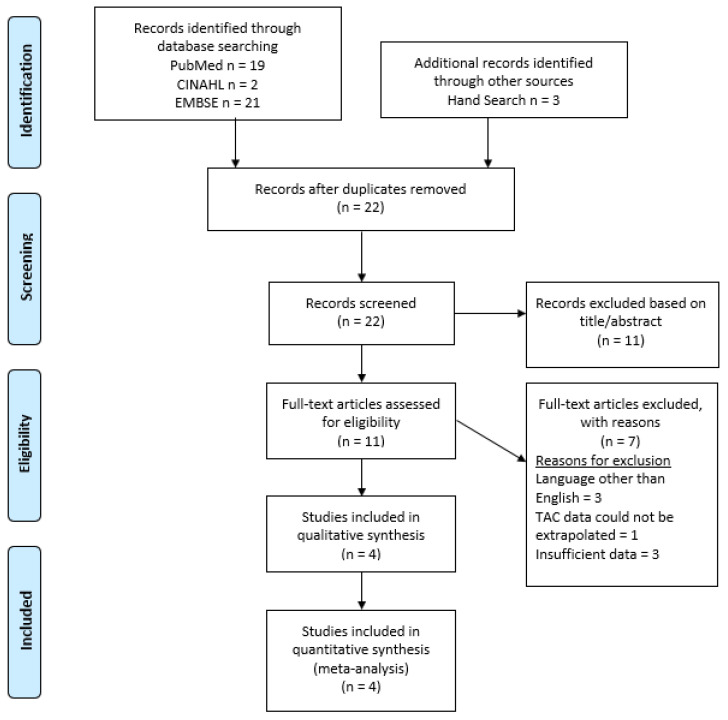
Prisma flow diagram.

The meta-analysis was conducted according to the Preferred Reporting Items for Systematic Reviews and Meta-Analyses (PRISMA) standards [25]. See Figure 1 for the Prisma Flow Diagram. An Institutional Review Board application was submitted at the University of Iowa and deemed to be non-human subjects research and exempt from IRB review.

### 2.1. Search Strategy

A literature search was performed by a health sciences librarian on the following electronic databases: MEDLINE via PubMed interface, EMBASE, and CINAHL (Cumulative Index to Nursing and Allied Health Literature), over a two-week span from May to June 2019 and re-run in February 2022, starting with PubMed and progressing in order as listed. The search terms were developed by the primary investigator and the health sciences librarian, who combined the terms to create a comprehensive search strategy. The original search strategy was then adapted by the health sciences librarian for the other databases listed. All databases were searched without the use of any filters, including language restrictions.

An example of a portion of the search utilized in PubMed follows, and can be viewed in full in the supplemental files: “Abnormalities, Multiple”[Mesh] OR Multiple Abnormalities [tw] OR “Anodontia”[Mesh] OR Anodontia [tw] OR Familial Tooth Ageneses [tw] OR Familial Tooth Agenesis [tw] OR Hypodontia Oligodontia 1 [tw] OR Hypodontia Oligodontia 1 s [tw] OR Hypodontia [tw] OR “Cleft Lip”[Mesh] OR Cleft Lip [tw] OR Cleft Lips [tw] OR Harelip [tw] OR Harelips [tw] AND Tooth Agenesis Code [tw] OR Tooth Agenesis Code TAC [tw]. Full search strategies for PubMed, CINAHL, and EMBASE can be found in Supplementary Figures S1, S2, and S3, respectively.

### 2.2. Eligibility and Exclusion Criteria

The eligibility criteria were defined before the literature search and are as follows: studies with (1) original data evaluating tooth agenesis, (2) Tooth Agenesis Code (TAC) used to evaluate patterns of tooth agenesis, (3) reporting full mouth TAC results or could be interpreted or attained from authors, (4) statistically compared tooth agenesis, (5) subjects with non-syndromic OFC, (6) non-syndromic subjects without OFC, (7) agenesis of 1–5 teeth (hypodontia), and (8) permanent dentition. Exclusion criteria include: (1) subjects with oligodontia, (2) syndromic OFC, or (3) other syndromes or diseases. Methods papers, reviews, commentaries, case reports, case series, and editorials were included.

### 2.3. Study Selection

Titles and abstracts were evaluated for all articles found in the database searches after duplicates were removed. If eligibility could not be determined from the title or abstract, evaluation of the full text was completed to determine eligibility, based on the previously defined criteria. All studies determined to be eligible were further evaluated and prepared for data extraction. Reviewers (B.H., X.X., and C.P.) agreed upon all studies included by consensus.

### 2.4. Data Collection

Data from the eligible articles were extracted to include, when present: publication date, journal, title, author(s), gender, age, presence of OFC and/or control group, dentition type (primary or permanent), full mouth TAC scores, cleft laterality, and cleft type.

One of the eligibility criteria was that the study had to use the TAC program (www.toothagenesiscode.com accessed from 1 October to 1 December 2019) to identify patterns in tooth agenesis for the permanent dentition. If studies reported full mouth TAC patterns, or patterns could be established from the data presented, or attained from the authors, they were included in the meta-analysis. TAC uses a binary coding notation where 0 = present and 1 = agenesis for each tooth in the mouth to identify patterns of agenesis. If a tooth has agenesis (binary code of 1), then each tooth is assigned a specific value which is calculated by 2^(n−1)^, where *n* = the tooth number (1–8) in each quadrant. Tooth 1 is the central incisor and tooth 8 is the third molar (Table 1). If no agenesis is present (binary code = 0), then that tooth is not assigned a specific value. See Figure 1 for a schematic representation of the unique values assigned for each tooth. For detailed methods regarding the program, please see van Wijk and Tan’s work [17]. The FDI tooth numbering system was utilized for this analysis. 

Unpublished TAC data on tooth agenesis in subjects with OFC from the OFC1 case cohort (*n* = 914 (623 with cleft lip and palate, 165 cleft lip, and 124 with cleft palate)) and OFC1 controls (*n* = 932) were included in the meta-analysis. These subjects were previously recruited from 11 international sites, including Colorado, Iowa, Texas, Puerto Rico, and Pennsylvania in the United States and internationally from Colombia, Guatemala, Hungary, Nigeria, Patagonia, and the Philippines. These subjects were scored for dental anomalies, including tooth agenesis, from in-person dental exams and/or intra-oral photos. The intra-rater and inter-rater reliability were excellent, with kappa ranging from 0.91 to 0.95. The Tooth Agenesis Code program was accessed between October and December 2019. For detailed methods, please refer to Howe et al.’s study [5].

### 2.5. Meta-Analysis

The overall TAC pattern frequency of each study was calculated as well as the frequencies broken down by gender for the studies where that was available (Hermus et al. 2013 [14], OFC1 case, and OFC1 control) and by laterality(Hermus et al. 2013 [14], OFC1 case, Lopez-Gimenez et al. 2018 [15], and Bartzela et al. 2013 [18]). The most common TAC patterns were selected for further analysis using a meta-analysis model and/or the Cochran–Mantel–Haenszel (CMH) test.

A random effects meta-analysis model was fit to estimate the proportion of each TAC pattern across all studies. The inverse of the variance of each study was utilized to determine how much weight to give each study in the model. This weighting scheme places more weight on studies with smaller variance. The meta-analysis model was run three times for each common overall TAC pattern. The first time included all datasets. The second time included only studies with a sample population that did not have CL/P (Souza-Silva et al. and OFC1 controls), and the third time included only studies with a sample population that have CL/P (Hermus et al. 2013 [14], Lopez-Gimenez et al. 2018 [15], and OFC1 cases). Since agenesis is much less prevalent in the populations that did not have CL/P, these three models were run to see how the proportions changed when looking at only those with CL/P and only those without CL/P vs. combining those with and without CL/P. Since two studies that have populations with CL/P (Hermus et al. [14] and OFC1 cases) and one study that had a population without CL/P (OFC1 control) provided sex information, the meta-analysis model was run twice for each sex. The first model utilized males or females from all three studies, while the second model used males or females from only studies with populations that had CL/P. Finally, nine meta-analysis models were run for each TAC pattern when trying to determine the prevalence of patterns by cleft type and laterality, broken into nine categories: all CL, all CLP, left CL only, right CL only, bilateral CL only, left CLP, right CLP, bilateral CLP, and CP only.

Exploratory analysis was conducted by utilizing the CMH test to calculate a common odds ratio across the three datasets that provided gender information and determine if there were any statistically significant differences in the odds of males or females having a specific TAC pattern. The CMH test was also used to analyze two (Hermus et al. and OFC1 case) of the three studies that provided laterality information to determine if there were any statistically significant differences in the odds of having a specific TAC pattern based on laterality of the cleft. The Bartzela et al. 2013 [18] study could not be utilized when conducting the CMH test because that study only had bilateral cleft lip and palate cases, and thus no odds ratio could be calculated. Finally, the case and control data from the OFC1 dataset were compared using a Fisher’s exact test to determine if there was a significant difference in the odds of having a particular TAC pattern based on CL/P and non-CLP status. A *p*-value of 0.05 was selected as the threshold for significance, and no adjustments were made for multiple comparisons.

## 3. Results

### 3.1. Study Selection

A total of 42 articles were retrieved from the databases with an additional 3 articles found with hand searching and cross-referencing. After duplicates were removed, 22 articles remained. The abstracts and titles were reviewed to establish inclusion to review full-text articles. Of the 22 reviewed, 11 articles were discarded as they clearly did not meet the criteria. The full texts of the remaining 11 articles were reviewed for inclusion. Upon review of the full texts, an additional 7 were removed due to the following reasons: (a) no English version available (*n* = 3), (b) data did not include overall TAC and could not be extrapolated (*n* = 1), and (c) data insufficient to be included for analysis (*n* = 3). The remaining four articles were carefully evaluated and included in the meta-analysis [13,14,15,16]. All studies excluded third molars from their TAC analysis; therefore, third molars were also removed from the TAC analysis for the unpublished TAC data. The Prisma diagram can be found in Figure 1.

### 3.2. Study Characteristics

The study characteristics of the four articles included plus the unpublished data are presented in Table 2. The studies included were published between 2010 and 2018. The methods paper for the TAC was published in 2006 [17]. The journals included were: *European Journal of Oral Sciences*, *Odontology*, and *Archives in Oral Biology*. The sample sizes ranged between 118 and 914 for subjects with OFC, with a total of 2182 subjects, and between 932 and 2239 for control subjects, with a total of 3171. The samples, including the unpublished data, originated internationally from Brazil, Colombia, Guatemala, Hungary, The Netherlands, Nigeria, Argentina, the Philippines, and Spain, and from the United States, from Colorado, Iowa, Texas, Pennsylvania, and Puerto Rico. Ages, for all subjects, ranged from 8 to older than 17.5 years old. The prevalence of permanent tooth agenesis ranged from 29.8% to 60% in subjects with OFC and from 2.9% to 3.6% for control subjects.

### 3.3. TAC Patterns

**Figure 2:**
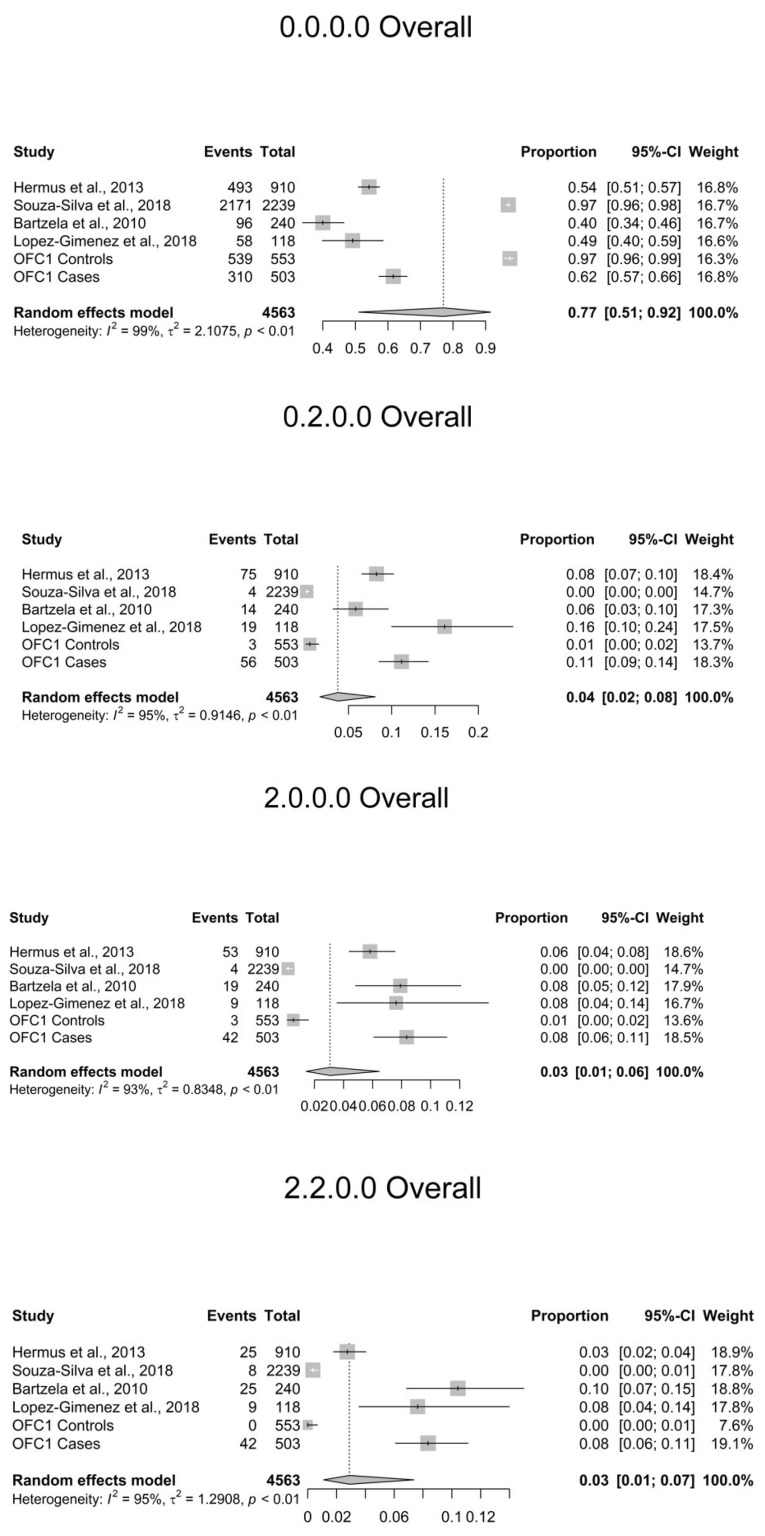
Forest plots of most common TAC patterns, all subjects [14,15,16,18].

The heterogeneity (I^2^) across the studies ranged from 0 to 99%. Subjects with no agenesis (0.0.0.0) comprised 97.1% of the combined control subjects and 51.6% of the combined subjects with OFC. A total of 31 TAC patterns, including no agenesis, from all studies combined were identified (Table 3). The most common TAC patterns seen in the combined groups were 0.2.0.0 (agenesis of tooth #22) (3.8%), 2.0.0.0 (agenesis of tooth #12) (3.1%), and 2.2.0.0 (agenesis of #12, 22) (2.9%). When broken down by case (OFC) and control, respectively, the most common TAC patterns for agenesis were 0.2.0.0 (9.7%/0.3%), 2.0.0.0 (7.1%/0.3%), 2.2.0.0 (6.6%/0.3%), and 0.16.0.0 (1.2%/0.1%). In examining the OFC1 dataset alone, which is the only dataset with both controls and cases, we found that subjects with OFC have statistically significant greater odds of having a TAC pattern with agenesis (*p* < 2.0 × 10^−16^). The results also suggest that subjects with OFC have statistically significant greater odds of having the following agenesis patterns: 0.2.0.0 (*p* = 1.3 × 10^−15^), 2.2.0.0 (1.2 × 10^−14^), and 2.0.0.0 (*p* = 3.3 × 10^−11^) (Table 4). TAC patterns including posterior teeth were not found to be significantly different between cases and controls overall or by cleft type. Therefore, the rate of posterior TA in OFC is consistent with that of controls and may indicate that OFC-related TA primarily affects the maxillary anterior teeth. Forest plots for the most common TAC patterns seen can be found in Figure 2.

### 3.4. Sex

In our analysis of TAC patterns by sex for combined studies (Hermus et al. and OFC1 dataset, as they had complete gender information for TAC patterns), based on the common odds ratios and *p*-values, there was no significant difference in the odds of males or females to have a particular TAC pattern. The most common TAC patterns in males were no agenesis 0.0.0.0 (78%), 0.2.0.0 (7.4%), 2.0.0.0 (5.1%), and 2.2.0.0 (2.8%). In females, the most common TAC patterns included no agenesis 0.0.0.0 (80%), 0.2.0.0 (4.9%), 2.0.0.0 (3.9%), and 2.2.0.0 (3.5%). Interestingly, TAC 16.0.0.0 (agenesis of #15) was not observed in any female subjects and was only observed in males (both case and control) (Supplementary Tables S1 and S2).

### 3.5. Cleft Type

The meta-analysis suggests that cleft type may have an impact on the frequency of certain TAC patterns. A subject with a cleft lip and palate (CLP) has greater odds of having a TAC pattern with agenesis when compared to a subject with a cleft lip (CL) (OR = 2.8, *p* = 6.4 × 10^−14^). When examining individual agenesis patterns, subjects with CLP were found to have the TAC patterns 0.2.0.0 and 2.2.0.0 significantly more often than CL (OR = 0.49, *p* = 0.0041, and OR = 0.40, *p* = 0.029, respectively, by pattern) and TAC patterns 0.2.0.0, 2.0.0.0, and 2.2.0.0 significantly more often than subjects with CP (OR = 13.1, *p* = 9.3 × 10^−8^, OR = 9.2, *p* = 2.1 × 10^−5^, and OR = 3.1, *p* = 0.02, respectively, by pattern). Subjects with CL only were more likely to have TAC patterns 0.2.0.0 and 2.0.0.0 compared to subjects with CP only (OR = 6.1, *p* = 0.0017, and OR = 4.4, *p* = 0.016, respectively). Interestingly, pattern 0.0.0.16 was more common in CL only compared to CLP (OR = 13.3, *p* = 0.045). See Table 5 for all cleft type results.

### 3.6. Laterality

When examining laterality (Right, Left, Bilateral) by cleft type, several TAC patterns were significantly more prevalent, including 0.2.0.0, 2.0.0.0, and 2.2.0.0. In individuals with CL, TAC pattern 2.0.0.0 is more likely to occur with right CL (*p* = 0.002) as compared to left CL, and pattern 2.2.0.0 is more likely to occur with bilateral CL as compared to right or left CL (*p* = 0.018 and *p* = 0.0001, respectively). No other patterns were significant for laterality with CL (Supplementary Table S3). This indicates that TA tends to be ipsilateral to the side of the cleft, with bilateral clefts having bilateral TA more often.

In cleft lip and palate, several patterns showed significance. Pattern 0.2.0.0 was more likely to occur in left CLP compared to right CLP or bilateral CLP (*p* = 1.0 × 10^−5^ and *p* = 0.0014, respectively). Individuals with right CLP are more likely to have pattern 2.0.0.0 when compared with left CLP (*p* = 7.6 × 10^−7^). Pattern 2.0.0.0 also occurs more frequently in right CLP (*p* = 0.0006) compared to BCLP. Pattern 2.2.0.0 was found to occur more often in BCLP when compared to right CLP (*p* = 0.019). See Supplementary Table S4. No TAC patterns including posterior teeth were found to be significant.

In comparing laterality and cleft type, an individual with left CLP is more likely to have an agenesis pattern (*p* = 4.5 × 10^−8^) compared to left CL. Pattern 0.2.0.0 is also more likely in left CLP (*p* = 0.006) when compared to left CL. When examining agenesis patterns associated with right CL or right CLP, individuals with right CLP are more likely to have an agenesis pattern (*p* = 9.9 × 10^−5^) compared to right CL. Pattern 2.0.0.0 was trending (*p* = 0.06) as more likely in right CLP when compared to right CL. See Supplementary Tables S3–S6.

## 4. Discussion

This meta-analysis represents a comprehensive examination of the current literature of the TAC program and TA in subjects with non-syndromic OFC and controls. Overall, permanent TA, excluding third molars, occurred at rates of 46% for subjects with OFC and 3% for control subjects. These rates are consistent with previously published studies of subjects with OFC (9.3–40.5%) and controls (0.6–5.2%) [1,5,6,8,26]. Among those with TA, 30 TAC patterns were uncovered, ranging in prevalence from 0.1% to 0.38% in controls to 0.1% to 9.7% in individuals with OFC. In the control populations [5,16], the most common TAC patterns were 0.2.0.0 (0.3%), 2.0.0.0 (0.3%), 2.2.0.0 (0.3%), and 0.0.0.16 (0.2%), with all others (26) falling below a 0.20% rate of occurrence. These same TAC patterns were found in individuals with OFC but at a higher rate of occurrence, 0.2.0.0 (9.7%), 2.0.0.0 (7.1%), 2.2.0.0 (6.6%), and 0.16.0.0 (1.2%), suggesting that, although the TA occurred in the same teeth, the etiology of TA may be due to the cleft itself or a complication of the surgical intervention and not genetics alone.

The results from the meta-analysis are further supported by looking only at the OFC1 dataset, as it is the only dataset in this analysis that has cases and controls in the same study, and also a recent publication that although not included in our analyses due to its recent appearance, we discussed here [27]. We found similar TAC patterns as compared to all studies included in the meta-analysis, with 0.2.0.0, 2.0.0.0, and 2.2.0.0 being significant when comparing cases vs. controls. Hermus and Lopez-Gimenez found the TAC pattern 0.2.0.0 to be the most common, with 15.7% and 19.1%, respectively, with Bartzela finding it to be the third most common TAC pattern at a rate of 5.8%. These findings are consistent with the OFC1 dataset (11%) and the recent work by Konstantonis et al., 2022 [27]. These findings confirm that the maxillary lateral incisor is the most common tooth to have agenesis (excluding third molars) in subjects with OFC, with the definitive patterns revealing agenesis of no other tooth besides the maxillary lateral incisor alone (0.2.0.0 or 2.0.0.0) or together (2.2.0.0). No significant TAC patterns involving posterior teeth were found in subjects with OFC, regardless of cleft type or laterality, and there were no significant TAC patterns involving posterior teeth when comparing subjects with OFC to controls. In our previous study [5] (OFC1), we examined dental anomalies, including TA noted as binary (yes or no) irrespective of where the TA was located, and we found that subjects with OFC had significantly more TA when compared to controls. In the current TAC analysis, when comparing cases and controls of the OFC1 cohort, the TAC patterns revealed that the statistical significance of TA in the previous study (Howe et al. 2015) was mostly driven by maxillary lateral incisor TA and not posterior TA. This provides insight into the possible etiology of incisor TA vs. posterior teeth TA in orofacial clefting. These findings suggest that subjects with OFC do not have an increased risk of posterior TA overall compared to controls. This may indicate that TA in the maxillary anterior teeth in subjects with OFC is primarily due to the cleft itself or surgery and that posterior TA may be due to random genetic mutations or environmental factors similar to the general population.

Regarding cleft type, we found that the most common TAC patterns overall, 0.2.0.0 and 2.2.0.0, were seen significantly more in CLP compared to CL (*p* = 0.0041 and *p* = 0.029, respectively, by pattern). Likewise, TAC patterns 0.2.0.0, 2.0.0.0, and 2.2.0.0 occurred significantly more in CLP than in CP (*p* = 9.3 × 10^−8^, *p* = 2.1 × 10^−5^, and *p* = 0.02, respectively, by pattern). This is consistent among studies included in the meta-analysis [5,13,14] and suggests that the more severe the cleft, the more likely the individual is to have TA. Interestingly, TAC pattern 0.0.0.16 was found to be significantly more common in subjects with CL compared to CLP (*p* = 0.045). In examining laterality, TAC patterns of the maxillary lateral incisors were consistently associated with the ipsilateral side of the cleft regardless of cleft type (CLP, CL) and with TA occurring bilaterally (2.2.0.0) in BCLP. Genetic analysis of these subgroups, regarding cleft type and laterality, could provide insight into intra- and inter-group genetic commonalties or differences, thus shedding light on possible genetic pathways leading to TA.

Sex was not found to be significant regarding TAC patterns, except for 16.0.0.0, where more males were found with the pattern compared to females. Although, this result should be interpreted with caution because only two studies included in our meta-analyses (Hermus et al. 2013 and OFC1) had sex data for each pattern, and this is a limitation of this study. The most recent study by Konstantinos et al. also found no relation between gender and TAC patterns [27]. Another limitation of the meta-analysis is the lack of TAC studies with both case and control groups. Currently, only the OFC1 dataset included in this meta-analysis has both cases and controls. Additionally, most studies only included the permanent dentition, and future studies are needed to address agenesis patterns in the primary dentition, which will help to tease out etiological factors related to agenesis from corrective surgical procedures, the cleft itself, genetic risk, or environmental factors. This is currently underway with the OFC1 dataset. Publication bias may also be a limitation of this study and could lead to over-estimation of TAC patterns; however, this may have a limited impact on the results since the data and results are more descriptive in nature.

In conclusion, the current meta-analysis shows that individuals with OFC are more likely to exhibit specific TA patterns compared with controls regarding both specific patterns as well as cleft type and cleft laterality. This study also suggests that TA in posterior teeth may not be due to the cleft itself or an increased genetic risk in subjects with OFC and that the risk for posterior TA is no different from controls. However, more case-control designed studies of subjects with OFC and controls using the TAC program to evaluate agenesis patterns are needed in both the primary and permanent dentitions. Further defining the phenotypic patterns of tooth agenesis in subjects with OFC vs. controls in both the primary and permanent dentition will help to increase our understanding of the complex etiological factors affecting tooth agenesis. Gaining a greater phenotypic understanding of agenesis patterns and pairing them with specific genetic data from individuals exhibiting these patterns could allow researchers to examine groups of subjects with the same agenesis pattern, giving us greater insight into phenotype–genotype relationships, and could allow targeted genetic studies bases on phenotypes.

## Figures and Tables

**Table 1:** Schematic representation of the dentition to determine Tooth Agenesis Code values.

	Maxillary Right (Q1)							Maxillary Left (Q2)						
Maxillary	17	16	15	14	13	12	11	21	22	23	24	25	26	27
AV	64	32	16	8	4	2	1	1	2	4	8	16	32	64
Mandibular	47	46	45	44	43	42	41	31	32	33	34	35	36	37
	Mandibular Right (Q4)							Mandibular Left (Q3)						

Note: AV = value associated with missing tooth due to agenesis. Teeth are numbered using the FDI system. Q1, Q2, Q3, and Q4 are quadrants 1–4. Tooth number 8 (18, 28, 38, 48) was not included in the study or in the schematic.

**Table 2:** Summary of included articles and unpublished data on Tooth Agenesis Code (TAC) [14,15,16,18].

Study Title	Authors	Publication Date	Control Group	Outcome Assessed	Sample Size	Sample	Sex Provided
Tooth agenesis patterns in bilateral cleft lip and palate [18]	Theodosia N. Bartzela, Carine E.L. Carels, Ewald M. Bronkhorst, Elisabeth Rønning, Sara Rizell, Anne Marie Kuijpers-Jagtman	2010	No	TAC patterns	240	CL/P	No
Patterns of tooth agenesis in patients with orofacial clefts [14]	Ruurd R. Hermus, Arjen J. van Wijk, Stephan P. K. Tan, Gem J. C. Kramer, Edwin M. Ongkosuwito	2013	No	TAC patterns	910	CL/P	Yes
Tooth agenesis code (TAC) in complete unilateral and bilateral cleft lip and palate patients [15]	Ana López-Giménez, Javier Silvestre-Rangil, Francisco Javier Silvestre, Vanessa Paredes-Gallardo	2017	No	TAC patterns	118	CL/P	No
Non-syndromic tooth agenesis patterns and their association with other dental anomalies: A retrospective study [16]	Bianca Núbia Souza-Silva, Walbert de Andrade Vieira, Ítalo de Macedo Bernardino, Marília Jesus Batistad, Marcos Alan Vieira Bittencourt, Luiz Renato Paranhos	2018	No	TAC patterns	2239	Non-CL/P	No
OFC1 Data	Unpublished		Yes	TAC patterns	1056	CL/P and Non-CL/P	Yes

**Table 3:** TAC patterns for case, control, and combined meta-analysis data.

	Combined Data Proportion (95% CI)	I^2^: Combined	Case Data Proportion (95% CI)	I^2^: Cases	Control Data Proportion (95% CI)	I^2^: Controls
0.0.0.0	0.77 (0.51, 0.915)	99.36%	0.516 (0.432, 0.599)	90.4%	0.971 (0.964, 0.976)	0%
0.2.0.0	0.038 (0.017, 0.081)	95.06%	0.097 (0.07, 0.135)	76.37%	0.003 (0.001, 0.009)	52.85%
2.0.0.0	0.031 (0.014, 0.065)	93.43%	0.071 (0.058, 0.086)	19.07%	0.003 (0.001, 0.009)	52.85%
2.2.0.0	0.029 (0.011, 0.074)	95.12%	0.066 (0.035, 0.119)	89.54%	0.003 (0.002, 0.006)	0%
0.16.0.0	0.005 (0.002, 0.015)	71.07%	0.012 (0.005, 0.025)	44.28%	0.001 (0, 0.006)	31.51%
0.0.16.16	0.003 (0.001, 0.011)	66.22%	0.009 (0.004, 0.018)	15.08%	0.001 (0, 0.004)	0%
0.0.0.16	0.005 (0.002, 0.009)	37.02%	0.007 (0.004, 0.013)	0%	0.002 (0.001, 0.005)	0%
16.16.0.0	0.004 (0.001, 0.012)	71.33%	0.008 (0.003, 0.021)	51.68%	0.001 (0, 0.003)	0%
16.16.16.16	0.003 (0.001, 0.007)	46.54%	0.006 (0.003, 0.012)	0%	0.001 (0, 0.004)	0%
0.0.16.0	0.004 (0.002, 0.01)	59.28%	0.008 (0.004, 0.015)	14.71%	0.001 (0, 0.003)	0%
16.0.0.0	0.005 (0.002, 0.012)	54.64%	0.008 (0.005, 0.014)	0%	0.001 (0, 0.006)	31.51%
0.18.0.0	0.003 (0.001, 0.009)	50.54%	0.006 (0.002, 0.013)	18.98%	0 (0, 0.003)	0%
16.2.0.0	0.003 (0.001, 0.008)	51.36%	0.005 (0.002, 0.013)	30.72%	0 (0, 0.003)	0%
2.16.0.0	0.003 (0.001, 0.011)	68.77%	0.005 (0.001, 0.02)	66.83%	0 (0, 0.003)	0%
0.0.2.0	0.002 (0.001, 0.005)	0%	0.004 (0.002, 0.009)	0%	0.001 (0, 0.004)	0%
0.1.0.0	0.003 (0.001, 0.006)	15.53%	0.004 (0.002, 0.009)	0%	0.001 (0, 0.003)	0%
0.2.16.0	0.002 (0.001, 0.008)	54.41%	0.004 (0.001, 0.015)	50.02%	0 (0, 0.003)	0%
16.18.16.16	0.002 (0.001, 0.006)	23.5%	0.004 (0.002, 0.009)	0%	0 (0, 0.003)	0%
18.0.0.0	0.002 (0, 0.01)	71.05%	0.004 (0.001, 0.022)	71.06%	0 (0, 0.003)	0%
18.18.0.0	0.005 (0.001, 0.015)	72.51%	0.009 (0.003, 0.024)	65.58%	0 (0, 0.003)	0%
2.18.0.0	0.003 (0.001, 0.008)	51.66%	0.004 (0.002, 0.013)	36.91%	0 (0, 0.003)	0%
2.2.16.16	0.002 (0.001, 0.006)	24.42%	0.004 (0.002, 0.009)	0%	0.001 (0, 0.003)	0%
0.0.0.2	0.001 (0.001, 0.003)	0%	0.002 (0, 0.006)	0%	0.001 (0, 0.003)	0%
0.2.16.16	0.001 (0.001, 0.004)	0%	0.002 (0.001, 0.007)	0%	0 (0, 0.003)	0%
0.4.0.0	0.002 (0.001, 0.004)	0%	0.003 (0.001, 0.007)	0%	0.001 (0, 0.003)	0%
0.8.0.0	0.001 (0.001, 0.003)	0%	0.002 (0.001, 0.007)	0%	0.001 (0, 0.003)	0%
1.0.0.0	0.003 (0.001, 0.007)	43.62%	0.005 (0.002, 0.011)	0%	0.001 (0, 0.003)	0%
16.18.0.0	0.002 (0.001, 0.005)	15.81%	0.003 (0.001, 0.008)	0%	0 (0, 0.003)	0%
18.18.16.16	0.003 (0.001, 0.01)	67.84%	0.005 (0.001, 0.019)	62.8%	0 (0, 0.003)	0%
0.0.32.32	0.001 (0, 0.003)	0%	0.001 (0, 0.006)	0%	0.001 (0, 0.004)	0%
1.1.0.0	0.001 (0.001, 0.004)	4.2%	0.002 (0.001, 0.008)	2.78%	0.001 (0, 0.003)	0%

Note: Proportions (95% CI).

**Table 4:** Comparison of OFC1 case and control data.

	Control Percent with Pattern	Case Percent with Pattern	Odds Ratio (95% CI)	*p*-Value
0.0.0.0	97.47%	61.63%	23.91 (13.6, 45.38)	<2 × 10^−16^
0.2.0.0	0.54%	11.13%	0.04 (0.01, 0.14)	1.3 × 10^−15^
2.0.0.0	0.54%	8.35%	0.06 (0.01, 0.19)	3.3 × 10^−11^
2.2.0.0	0%	8.35%	0 (0, 0.08)	1.2 × 10^−14^
0.16.0.0	0.18%	0.2%	0.91 (0.01, 71.49)	>0.99
0.0.16.16	0.18%	0%	Inf (0.02, Inf)	>0.99
0.0.0.16	0%	0.4%	0 (0, 4.84)	0.23
16.16.0.0	0%	0.2%	0 (0, 35.47)	0.48
16.16.16.16	0.18%	0%	Inf (0.02, Inf)	>0.99
0.0.16.0	0.18%	0%	Inf (0.02, Inf)	>0.99
16.0.0.0	0.18%	0.6%	0.3 (0.01, 3.78)	0.35
0.18.0.0	0%	0%	0 (0, Inf)	>0.99
16.2.0.0	0%	0%	0 (0, Inf)	>0.99
2.16.0.0	0%	0.2%	0 (0, 35.47)	0.48
0.0.2.0	0%	0.2%	0 (0, 35.47)	0.48
0.1.0.0	0.18%	0.6%	0.3 (0.01, 3.78)	0.35
0.2.16.0	0%	0%	0 (0, Inf)	>0.99
16.18.16.16	0%	0.2%	0 (0, 35.47)	0.48
18.0.0.0	0%	0%	0 (0, Inf)	>0.99
18.18.0.0	0%	0.6%	0 (0, 2.2)	0.11
2.18.0.0	0%	0.2%	0 (0, 35.47)	0.48
2.2.16.16	0%	0.2%	0 (0, 35.47)	0.48
0.0.0.2	0.18%	0%	Inf (0.02, Inf)	>0.99
0.2.16.16	0%	0%	0 (0, Inf)	>0.99
0.4.0.0	0%	0.4%	0 (0, 4.84)	0.23
0.8.0.0	0%	0%	0 (0, Inf)	>0.99
1.0.0.0	0%	0.6%	0 (0, 2.2)	0.11
16.18.0.0	0%	0%	0 (0, Inf)	>0.99
18.18.16.16	0%	0.2%	0 (0, 35.47)	0.48
0.0.32.32	0.18%	0%	Inf (0.02, Inf)	>0.99
1.1.0.0	0%	0%	0 (0, Inf)	>0.99

Note: Proportions. OR = odds ratio (95% CI).

**Table 5:** Cleft type and TAC patterns.

	CL	I^2^	CLP	I^2^	CP	I^2^	CL vs. CLP OR	CL vs. CP OR	CLP vs. CP OR
0.0.0.0	0.693 (0.642, 0.74)	0%	0.45 (0.412, 0.488)	33.54%	0.879 (0.281, 0.993)	93.98%	2.829 (2.151, 3.722) *p* = 6.4 × 10^−14^	Failed BD Test	Failed BD Test
0.2.0.0	0.067 (0.037, 0.119)	27.35%	0.102 (0.064, 0.16)	83.6%	0.014 (0.005, 0.039)	0%	0.489 (0.303, 0.788) *p* = 0.0041	6.194 (1.84, 20.847) *p* = 0.0017	13.134 (4.07, 42.384) *p* = 9.3 × 10^−8^
0.0.16.16	0.012 (0.004, 0.034)	0%	0.006 (0.003, 0.014)	0%	0.028 (0.006, 0.116)	37.12%	Failed BD Test	Failed BD Test	Failed BD Test
0.1.0.0	0.025 (0.011, 0.059)	7.37%	0.004 (0.002, 0.011)	0%	0.004 (0.001, 0.029)	0%	8.528 (1.129, 64.421) *p* = 0.026	Failed BD Test	Failed BD Test
0.16.0.0	0.013 (0.005, 0.034)	0%	0.02 (0.012, 0.031)	0%	0.006 (0.001, 0.028)	0%	0.337 (0.074, 1.545) *p* = 0.24	Failed BD Test	4.477 (0.57, 35.156) *p* = 0.22
16.2.0.0	0.012 (0.004, 0.034)	0%	0.008 (0.004, 0.016)	0%	0.004 (0.001, 0.029)	0%	Failed BD Test	Failed BD Test	Failed BD Test
2.0.0.0	0.067 (0.029, 0.146)	61.14%	0.093 (0.059, 0.144)	80.34%	0.014 (0.005, 0.039)	0%	Failed BD Test	4.48 (1.321, 15.194) *p* = 0.016	9.221 (2.832, 30.029) *p* = 2.1 × 10^−5^
2.2.0.0	0.037 (0.011, 0.111)	61.61%	0.081 (0.05, 0.127)	72.74%	0.019 (0.008, 0.045)	0%	0.407 (0.189, 0.879) *p* = 0.029	1.239 (0.402, 3.823) *p* = 0.93	3.147 (1.227, 8.072) *p* = 0.02
0.0.0.16	0.021 (0.009, 0.049)	0%	0.006 (0.003, 0.014)	0%	0.023 (0.01, 0.052)	0%	13.304 (1.174, 150.774) *p* = 0.045	0.604 (0.161, 2.264) *p* = 0.68	Failed BD Test
0.16.16.16	0.013 (0.005, 0.036)	0%	0.006 (0.003, 0.013)	0%	0.004 (0.001, 0.029)	0%	Failed BD Test	Failed BD Test	Failed BD Test
16.0.0.0	0.012 (0.004, 0.036)	0%	0.012 (0.007, 0.022)	0%	0.01 (0.003, 0.033)	0%	Failed BD Test	Failed BD Test	1.206 (0.24, 6.07) *p* = 0.82
16.16.0.0	0.012 (0.004, 0.036)	0%	0.014 (0.008, 0.024)	0%	0.01 (0.003, 0.033)	0%	Failed BD Test	Failed BD Test	1.164 (0.23, 5.903) *p* = 0.86
16.18.16.16	0.012 (0.004, 0.036)	0%	0.007 (0.003, 0.015)	0%	0.004 (0.001, 0.029)	0%	Failed BD Test	Failed BD Test	Failed BD Test
0.0.16.0	0.012 (0.004, 0.036)	0%	0.006 (0.002, 0.013)	0%	0.023 (0.01, 0.052)	0%	Failed BD Test	Failed BD Test	Failed BD Test
0.18.0.0	0.012 (0.004, 0.036)	0%	0.011 (0.006, 0.02)	0%	0.004 (0.001, 0.029)	0%	Failed BD Test	Failed BD Test	Failed BD Test
16.16.16.16	0.012 (0.004, 0.036)	0%	0.006 (0.002, 0.013)	0%	0.014 (0.005, 0.039)	0%	Failed BD Test	Failed BD Test	Failed BD Test
2.2.16.16	0.012 (0.004, 0.036)	0%	0.007 (0.003, 0.014)	0%	0.004 (0.001, 0.029)	0%	Failed BD Test	Failed BD Test	Failed BD Test
2.16.0.0	0.012 (0.004, 0.036)	0%	0.008 (0.004, 0.018)	0%	0.004 (0.001, 0.029)	0%	Failed BD Test	Failed BD Test	Failed BD Test
1.0.0.0	0.012 (0.004, 0.036)	0%	0.009 (0.005, 0.019)	0%	0.004 (0.001, 0.029)	0%	Failed BD Test	Failed BD Test	Failed BD Test
3.0.0.0	0.012 (0.004, 0.036)	0%	0.006 (0.002, 0.014)	0%	0.004 (0.001, 0.029)	0%	Failed BD Test	Failed BD Test	Failed BD Test
18.18.0.0	0.012 (0.004, 0.036)	0%	0.014 (0.008, 0.024)	0%	0.004 (0.001, 0.029)	0%	Failed BD Test	Failed BD Test	Failed BD Test
2.18.0.0	0.012 (0.004, 0.036)	0%	0.009 (0.005, 0.018)	0%	0.004 (0.001, 0.029)	0%	Failed BD Test	Failed BD Test	Failed BD Test
18.18.16.16	0.012 (0.004, 0.036)	0%	0.01 (0.005, 0.02)	0%	0.004 (0.001, 0.029)	0%	Failed BD Test	Failed BD Test	Failed BD Test
0.0.2.0	0.012 (0.004, 0.036)	0%	0.006 (0.003, 0.013)	0%	0.01 (0.003, 0.033)	0%	Failed BD Test	Failed BD Test	0.125 (0.009, 1.821) *p* = 0.32
18.2.0.0	0.012 (0.004, 0.036)	0%	0.009 (0.004, 0.019)	0%	0.004 (0.001, 0.029)	0%	Failed BD Test	Failed BD Test	Failed BD Test

Note: Proportions (95% CI). OR = odds ratio.

## Data Availability

Data presented in this study are available upon request from the corresponding author for collaborative projects.

## Supplementary Materials

The following supporting information can be downloaded at: https://www.mdpi.com/article/10.3390/dj10070128/s1, Supplementary Figure S1: PubMed Search Criteria; Supplementary Figure S2: CINAHL Search Criteria; Supplementary Figure S3: EMBASE Search Criteria; Supplementary Table S1: Gender and TAC Patterns; Supplementary Table S2: Gender and TAC Patterns of OFC; Supplementary Table S3: TAC Patterns and Cleft Lip Only and Laterality; Supplementary Table S4: TAC Patterns and Cleft Lip and Palate and Laterality; Supplementary Table S5: TAC Patterns and Left Cleft Lip vs. Cleft Lip and Palate; Supplementary Table S6: TAC Patterns and Right Cleft Lip vs. Cleft Lip and Palate.
