# Supplementary material for: Tooth Agenesis Patterns in Orofacial Clefting Using Tooth Agenesis Code: A Meta-Analysis
Source: Dent J (Basel). 2022 Jul 5;10(7):128. doi: 10.3390/dj10070128 (PMC9323030; doi:10.3390/dj10070128)
Supplement: Supplementary file 1 [file dentistry-10-00128-s001.zip › dentistry-1707078-supplementary.pdf]

Supplemental Figure S1. PubMed Search Strategy

"Abnormalities, Multiple"[Mesh] OR Multiple Abnormalities [tw] OR **"Anodontia"[Mesh]** OR Anodontia [tw] OR Familial Tooth Ageneses [tw] OR Familial Tooth Agenesis [tw] OR Hypodontia Oligodontia 1 [tw] OR Hypodontia Oligodontia 1s [tw] OR Hypodontia [tw] OR **"Cleft Lip"[Mesh]** OR Cleft Lip [tw] OR Cleft Lips [tw] OR Harelip [tw] OR Harelips [tw] OR **"Cleft Lip with or without Cleft Palate, Nonsyndromic, 8" [Supplementary Concept]** OR Orofacial Cleft 8 [tw] OR **"Cleft Palate"[Mesh]** OR Cleft Palate [tw] OR Cleft Palates [tw] OR Isolated Cleft Palate [tw] OR Isolated Cleft Palates [tw] OR **"Ecotype"[Mesh]** OR Ecotype [tw] OR Ecotypes [tw] OR **"Gene-Environment Interaction"[Mesh]** OR Gene-Environment Interaction [tw] OR Gene Environment Interaction [tw] OR Gene-Environment Interactions [tw] OR Environment-Gene Interaction [tw] OR Environment Gene Interaction [tw] OR Environment-Gene Interactions [tw] OR **"Mandible"[Mesh]** OR Mandible [tw] OR Mandibles [tw] OR Mylohyoid Ridge [tw] OR Mylohyoid Ridges [tw] OR Mylohyoid Groove [tw] OR Mylohyoid Grooves [tw] OR **"Maxilla"[Mesh]** OR Maxilla [tw] OR Maxillas [tw] OR Maxillary Bone [tw] OR Maxillary Bones [tw] OR Maxillae [tw] OR **"Orofacial Cleft 1" [Supplementary Concept]** OR Orofacial Cleft 1 [tw] OR Nonsyndromic Cleft Lip-Palate [tw] OR Nonsyndromic Orofacial Cleft [tw] OR OFC1 [tw] OR **"Palate"[Mesh]** OR Palate [tw] OR Palates [tw] OR Incisive Papilla [tw] OR Incisive Papillas [tw] OR **"Palate, Hard"[Mesh]** OR Hard Palate [tw] OR Hard Palates [tw] OR Palatine Bone [tw] OR **"Palatal Muscles"[Mesh]** OR Palatal Muscles [tw] OR Palatal Muscle [tw] OR **"Palate, Soft"[Mesh]** OR Soft Palate [tw] OR Velum Palatinum [tw] OR **"Penetrance"[Mesh]** OR Penetrance [tw] OR **"Phenotype"[Mesh]** OR Phenotype [tw] OR **"Prevalence"[Mesh]** OR Prevalence [tw] OR Prevalences [tw] OR "**Tooth Agenesis, Selective, With Orofacial Cleft [Supplementary Concept]"** OR Tooth Agenesis, Selective, With Orofacial Cleft [tw] OR **"Tooth Loss"[Mesh]** OR Tooth Loss [tw] OR **"Uvula"[Mesh]** OR Uvula [tw] OR Agenesis* [tw] OR Agenesis Pattern* [tw] OR Bilateral Cleft Lip and Palate [tw] OR Cleft lip and Palate [tw] OR Complete Bilateral Cleft Lip and Palate [tw] OR Complete Unilateral Cleft Lip and Palate [tw] OR Congenitally missing teeth [tw] OR CUCLP [tw] OR Dental Agenesis [tw] OR Human Tooth Agenesis [tw] OR Nonsyndromic Oligodontia [tw] OR Nonsyndromic Orofacial Clefting [tw] OR Oligodontia [tw] OR Oligodontia Phenotype [tw] OR Orofacial cleft [tw] OR Permanent Tooth Agenesis [tw] OR Tooth Agenesis [tw] OR Unilateral Cleft Lip and Palate [tw]

AND

Group 2: Tooth Agenesis Code (TAC)

Tooth Agenesis Code [tw] OR Tooth Agenesis Code TAC [tw]

Supplemental Figure S2. CINAHL Search Strategy

Group 1: Agenesis Patterns in Nonsyndromic Orofacial Clefting

MH "Abnormalities, Multiple+" OR "Multiple Abnormalities" OR MH "Anodontia+" OR "Anodontia" OR "Familial Tooth Ageneses" OR "Familial Tooth Agenesis" OR "Hypodontia Oligodontia 1" OR "Hypodontia Oligodontia 1s" OR "Hypodontia" OR MH "Cleft Lip" OR "Cleft Lip" OR "Cleft Lips" OR "Harelip" OR "Cleft Lip with or without Cleft Palate, Nonsyndromic, 8" OR "Orofacial Cleft 8" OR MH "Cleft Palate" OR "Cleft Palate" OR "Cleft Palate" OR "Cleft Palates" OR "Isolated Cleft Palate" OR "Isolated Cleft Palates" OR "Ecotype" OR "Ecotypes" OR "Gene Environment Interaction" OR "Gene-Environment Interactions" OR "Environment Gene Interaction" OR "Environment-Gene Interactions" OR MH "Mandible+" OR "Mandible" OR "Mandibles" OR "Mylohyoid Ridge" OR "Mylohyoid Groove" OR MH "Maxilla" OR "Maxilla" OR "Maxillas" OR "Maxillary Bone" OR "Maxillary Bones" OR "Maxillae" OR "Orofacial Cleft 1" OR "Nonsyndromic Cleft Lip-Palate" OR "Nonsyndromic Orofacial Cleft" OR "OFC1" OR MH "Palate+" OR "Palate" OR "Palates" OR "Incisive Papilla" OR "Incisive Papillas" OR "Hard Palate" OR "Hard Palates" OR "Palatine Bone" OR MH "Palatal Muscles" OR "Palatal Muscles" OR "Palatal Muscle" OR (MH "Palate, Soft+") OR "Soft Palate" OR "Penetrance" OR MH "Phenotype+" OR "Phenotype" OR MH "Prevalence" OR "Prevalence" OR "Prevalences" OR "Tooth Agenesis, Selective, With Orofacial Cleft" OR (MH "Tooth Loss") OR "Tooth Loss" OR MH "Uvula" OR "Uvula" OR "Agenesis" OR "Ageneses" OR "Agenesis Pattern" OR "Agenesis Patterns" OR "Bilateral Cleft Lip and Palate" OR "Cleft lip and Palate" OR "Complete Bilateral Cleft Lip and Palate" OR "Complete Unilateral Cleft Lip and Palate" OR "Congenitally missing teeth" OR "CUCLP" OR "Dental Agenesis" OR "Human Tooth Agenesis" OR "Nonsyndromic Oligodontia" OR "Nonsyndromic Orofacial Clefting" OR "Oligodontia" OR "Oligodontia Phenotype" OR "Orofacial cleft" OR "Permanent Tooth Agenesis" OR "Tooth Agenesis" OR "Unilateral Cleft Lip and Palate"

AND

Group 2: Tooth Agenesis Code (TAC)

"Tooth Agenesis Code"

Supplemental Figure S3. EMBASE Search Strategy

Group 1: Agenesis Patterns in Nonsyndromic Orofacial Clefting

'multiple malformation syndrome'/exp OR 'multiple malformation syndrome':ti,ab OR 'multiple abnormalities':ti,ab OR **'anodontia'/exp** OR 'adontia':ti,ab OR 'anodontia':ti,ab OR 'familial tooth agenesis':ti,ab OR 'hypodontia':ti,ab OR **'cleft lip'/exp** OR 'cheiloschisis':ti,ab OR 'cleft lip':ti,ab OR 'cleft lip anatomy':ti,ab OR 'cleft lip embryology':ti,ab OR 'complete cleft lip':ti,ab OR 'congenital cleft lip':ti,ab OR 'hare lip':ti,ab OR 'harelip':ti,ab OR 'labioschisis':ti,ab OR 'labioschizis':ti,ab OR 'lip cleft':ti,ab OR 'cleft lips':ti,ab OR 'harelips':ti,ab OR **'cleft lip with or without cleft palate'/exp** OR 'cleft lip with or without cleft palate':ti,ab OR **'cleft palate'/exp** OR 'cleft maxilla palate':ti,ab OR 'cleft palate':ti,ab OR 'cleft palate classification':ti,ab OR 'cleft palate embryology':ti,ab OR 'cleft palatum':ti,ab OR 'palatal cleft':ti,ab OR 'palatischisis':ti,ab OR 'palatoschisis':ti,ab OR 'palatoschizis':ti,ab OR 'palatum fissum':ti,ab OR 'prepalatal cleft palate':ti,ab OR 'cleft palates':ti,ab OR 'isolated cleft palate':ti,ab OR 'isolated cleft palates':ti,ab OR **'ecotype'/exp** OR 'ecospecies':ti,ab OR 'ecotype':ti,ab OR 'ecovar':ti,ab OR 'ecotypes':ti,ab OR **'genotype environment interaction'/exp** OR 'environment-gene interaction':ti,ab OR 'gene environment interaction':ti,ab OR 'gene-environment interaction':ti,ab OR 'genotype environment interaction':ti,ab OR 'interaction, genotype environment':ti,ab OR 'gene-environment interactions':ti,ab OR **'mandible'/exp** OR 'lingula mandibulae':ti,ab OR 'lower jaw':ti,ab OR 'lower jaw lingula':ti,ab OR 'mandible':ti,ab OR 'mandible arch':ti,ab OR 'mandible lingula':ti,ab OR 'mandibula':ti,ab OR 'mandibular arch':ti,ab OR 'mandibular bone':ti,ab OR 'mandibular lingula':ti,ab OR 'mandibule':ti,ab OR 'mandibles':ti,ab OR 'mylohyoid ridge':ti,ab OR 'mylohyoid ridges':ti,ab OR 'mylohyoid groove':ti,ab OR 'mylohyoid grooves':ti,ab OR **'maxilla'/exp** OR 'maxilla':ti,ab OR 'maxillary':ti,ab OR 'maxillary area':ti,ab OR 'maxillary growth':ti,ab OR 'maxillofacial skeleton':ti,ab OR 'upper jaw':ti,ab OR 'maxillas':ti,ab OR 'maxillary bone':ti,ab OR 'maxillary bones':ti,ab OR 'maxillae':ti,ab OR 'orofacial cleft 1':ti,ab OR **'nonsyndromic cleft lip palate'/exp** OR 'nonsyndromic cleft lip palate':ti,ab OR 'nonsyndromic cleft lip-palate':ti,ab OR **'nonsyndromic orofacial cleft'/exp** OR 'nonsyndromic orofacial':ti,ab OR 'ofc1':ti,ab OR **'palate'/exp** OR 'palate':ti,ab OR 'palate defect':ti,ab OR 'palate lesion':ti,ab OR 'palatum':ti,ab OR 'palates':ti,ab OR 'incisive papilla':ti,ab OR **'hard palate'/exp** OR 'bony palate':ti,ab OR 'hard palate':ti,ab OR 'hard palatum':ti,ab OR 'palatum durum':ti,ab OR 'hard palates':ti,ab OR 'palatine bone':ti,ab OR **'palate muscle'/exp** OR 'palatal muscles':ti,ab OR 'palate muscle':ti,ab OR **'soft palate'/exp** OR 'palatine velum':ti,ab OR 'palatum molle':ti,ab OR 'soft palate':ti,ab OR 'velum palatinum':ti,ab OR **'penetrance'/exp** OR 'penetrance':ti,ab OR **'phenotype'/exp** OR 'phenotype':ti,ab OR 'phenotypic masking':ti,ab OR 'phenotyping':ti,ab OR **'prevalence'/exp** OR 'prevalence':ti,ab OR 'prevalence study':ti,ab OR 'prevalences':ti,ab OR **'periodontal disease'/exp** OR 'dental loss':ti,ab OR 'edentulism':ti,ab OR 'tooth loss':ti,ab OR 'tooth movement':ti,ab OR **'uvula'/exp** OR 'uvula':ti,ab OR **'agenesis'/exp** OR 'agenesia':ti,ab OR 'agenesis':ti,ab OR 'congenital absence':ti,ab OR 'congenital agenesis':ti,ab OR 'congenital bilateral absence':ti,ab OR **'bilateral cleft lip and palate'/exp** OR 'bilateral cleft lip and palate':ti,ab OR **'cleft lip palate'/exp** OR 'cleft lip and palate':ti,ab OR 'cleft lip palate':ti,ab OR 'cleft palate lip':ti,ab OR 'labiopalatoschisis':ti,ab OR 'palatolabioschisis':ti,ab OR 'complete bilateral cleft lip and palate':ti,ab OR 'complete unilateral cleft lip and palate':ti,ab OR 'congenitally missing teeth':ti,ab OR 'cuclp':ti,ab OR **'hypodontia'/exp** OR 'dental agenesis':ti,ab OR 'hypodontia':ti,ab OR 'partial anodontia':ti,ab OR 'tooth agenesia':ti,ab OR 'tooth agenesis':ti,ab OR 'human tooth agenesis':ti,ab OR **'oligodontia'/exp** OR 'oligodontia':ti,ab OR 'orofacial cleft'/exp OR 'orofacial cleft':ti,ab OR **'unilateral cleft lip and palate'/exp** OR 'unilateral cleft lip and palate':ti,ab OR

'nonsyndromic oligodontia':ti,ab OR 'nonsyndromic orofacial clefting':ti,ab OR 'oligodontia phenotype':ti,ab OR 'permanent tooth agenesis':ti,ab

AND

Group 2: Tooth Agenesis Code (TAC)

'tooth agenesis code':ti,ab OR 'tooth agenesis code (tac)':ti,ab

Supplemental Table S1. Gender and TAC pattern, all studies.

	Female Proportion (95% CI)	I^2	Male Proportion (95% CI	I^2	Common OR (95% CI)	P-Value
**0.0.0.0**	0.8 (0.541, 0.932)	97.53%	0.781 (0.572, 0.905)	97.36%	1.027 (0.831, 1.269)	0.85
**0.2.0.0**	0.049 (0.019, 0.124)	88.29%	0.074 (0.038, 0.138)	82.72%	0.834 (0.575, 1.208)	0.38
**2.0.0.0**	0.039 (0.016, 0.088)	75.97%	0.051 (0.024, 0.106)	85.42%	0.717 (0.464, 1.109)	0.16
**2.2.0.0**	0.035 (0.01, 0.114)	86.68%	0.028 (0.008, 0.092)	88.48%	Failed BD Test	NA
**0.16.0.0**	0.006 (0.002, 0.016)	0%	0.008 (0.002, 0.028)	55.18%	0.373 (0.106, 1.321)	0.18
**0.0.0.16**	0.005 (0.002, 0.013)	0%	0.007 (0.003, 0.018)	15.7%	Failed BD Test	NA
**16.0.0.0**	0.002 (0, 0.009)	0%	0.009 (0.005, 0.018)	0%	Failed BD Test	NA
**0.0.16.0**	0.004 (0.001, 0.012)	0%	0.006 (0.002, 0.013)	0%	Failed BD Test	NA
**16.16.16.16**	0.006 (0.002, 0.015)	0%	0.005 (0.002, 0.013)	0%	Failed BD Test	NA
**0.0.16.16**	0.007 (0.001, 0.044)	61.3%	0.004 (0.002, 0.011)	0%	Failed BD Test	NA
**0.18.0.0**	0.004 (0.001, 0.012)	0%	0.003 (0.001, 0.009)	0%	Failed BD Test	NA
**16.16.0.0**	0.007 (0.002, 0.027)	36.01%	0.003 (0.001, 0.009)	0%	Failed BD Test	NA
**16.2.0.0**	0.002 (0.001, 0.01)	0%	0.003 (0.001, 0.009)	0%	Failed BD Test	NA
**2.18.0.0**	0.003 (0.001, 0.011)	0%	0.003 (0.001, 0.009)	0%	Failed BD Test	NA
**0.1.0.0**	0.006 (0.002, 0.015)	0%	0.002 (0.001, 0.008)	0%	2.911 (0.516, 16.427)	0.41
**16.0.16.16**	0.006 (0.002, 0.016)	0%	0.002 (0, 0.007)	0%	Failed BD Test	NA
**2.16.0.0**	0.004 (0.001, 0.012)	0%	0.002 (0.001, 0.008)	0%	Failed BD Test	NA
**3.0.0.0**	0.002 (0, 0.009)	0%	0.004 (0.001, 0.011)	0%	Failed BD Test	NA
**0.0.2.0**	0.004 (0.001, 0.012)	0%	0.002 (0.001, 0.008)	0%	Failed BD Test	NA
**0.2.16.0**	0.004 (0.001, 0.012)	0%	0.001 (0, 0.007)	0%	Failed BD Test	NA
**0.6.0.0**	0.006 (0.003, 0.016)	0%	0.001 (0, 0.007)	0%	Failed BD Test	NA
**16.18.16.16**	0.004 (0.001, 0.012)	0%	0.002 (0.001, 0.008)	0%	Failed BD Test	NA
**18.18.0.0**	0.004 (0.001, 0.012)	0%	0.004 (0.001, 0.018)	37.93%	Failed BD Test	NA
**0.4.0.0**	0.002 (0.001, 0.01)	0%	0.004 (0.001, 0.012)	0%	Failed BD Test	NA
**1.0.0.0**	0.002 (0.001, 0.01)	0%	0.004 (0.001, 0.018)	37.93%	Failed BD Test	NA

Supplemental Table S2. Gender and TAC patterns, OFC subjects only.

	Female Proportion (95% CI)	I^2	Male Proportion (95% CI)	I^2
**0.0.0.0**	0.584 (0.485, 0.676)	80.2%	0.571 (0.513, 0.628)	63.71%
**0.2.0.0**	0.086 (0.048, 0.149)	76.06%	0.099 (0.081, 0.121)	0%
**2.0.0.0**	0.056 (0.04, 0.079)	0%	0.077 (0.051, 0.115)	66.22%
**2.2.0.0**	0.058 (0.02, 0.153)	88.1%	0.043 (0.013, 0.13)	91.67%
**0.16.0.0**	0.007 (0.002, 0.02)	0%	0.01 (0.002, 0.048)	62.38%
**0.0.0.16**	0.005 (0.002, 0.017)	0%	0.008 (0.003, 0.022)	18.07%
**16.0.0.0**	0.002 (0, 0.013)	0%	0.011 (0.005, 0.02)	0%
**0.0.16.0**	0.005 (0.001, 0.016)	0%	0.006 (0.002, 0.015)	0%
**16.16.16.16**	0.007 (0.002, 0.02)	0%	0.006 (0.002, 0.015)	0%
**0.0.16.16**	0.011 (0.002, 0.08)	56.77%	0.005 (0.002, 0.013)	0%
**0.18.0.0**	0.005 (0.001, 0.016)	0%	0.003 (0.001, 0.011)	0%
**16.16.0.0**	0.01 (0.003, 0.038)	27.29%	0.004 (0.001, 0.011)	0%
**16.2.0.0**	0.003 (0.001, 0.013)	0%	0.003 (0.001, 0.011)	0%
**2.18.0.0**	0.003 (0.001, 0.016)	0%	0.003 (0.001, 0.011)	0%
**0.1.0.0**	0.006 (0.002, 0.021)	7.53%	0.002 (0.001, 0.01)	0%
**16.0.16.16**	0.007 (0.002, 0.02)	0%	0.002 (0, 0.009)	0%
**2.16.0.0**	0.005 (0.001, 0.016)	0%	0.002 (0.001, 0.01)	0%
**3.0.0.0**	0.002 (0, 0.013)	0%	0.004 (0.001, 0.014)	11.13%
**0.0.2.0**	0.005 (0.001, 0.016)	0%	0.002 (0, 0.011)	0%
**0.2.16.0**	0.005 (0.001, 0.016)	0%	0.001 (0, 0.009)	0%
**0.6.0.0**	0.007 (0.003, 0.02)	0%	0.001 (0, 0.009)	0%
**16.18.16.16**	0.005 (0.001, 0.016)	0%	0.002 (0, 0.011)	0%
**18.18.0.0**	0.005 (0.001, 0.016)	0%	0.004 (0, 0.039)	59.61%
**0.4.0.0**	0.003 (0.001, 0.013)	0%	0.004 (0.001, 0.021)	37.2%
**1.0.0.0**	0.003 (0.001, 0.013)	0%	0.004 (0, 0.039)	59.61%

Supplemental Table S3. Cleft Lip Only and Laterality with TAC Patterns

	Left CL	I^2	Right CL	I^2	Bilateral CL	I^2	Left vs Right OR	Left vs Bilateral OR	Right vs Bilateral OR
**0.0.0.0**	0.727 (0.597, 0.827)	58.3%	0.696 (0.605, 0.774)	0%	0.632 (0.47, 0.768)	0%	1.057 (0.636, 1.757) P = 0.93	1.44 (0.694, 2.99) P = 0.43	1.334 (0.616, 2.89) P = 0.6
**0.2.0.0**	0.101 (0.065, 0.155)	1.86%	0.033 (0.006, 0.173)	54.89%	0.039 (0.008, 0.17)	0%	Failed BD Test	4.012 (0.518, 31.093) P = 0.27	1.068 (0.113, 10.097) P = 0.95
**0.0.16.16**	0.008 (0.002, 0.038)	0%	0.01 (0.001, 0.068)	0%	0.027 (0.004, 0.17)	0%	Failed BD Test	Failed BD Test	Failed BD Test
**0.1.0.0**	0.024 (0.003, 0.177)	72.51%	0.01 (0.001, 0.068)	0%	0.027 (0.004, 0.17)	0%	Failed BD Test	Failed BD Test	Failed BD Test
**0.16.0.0**	0.008 (0.002, 0.038)	0%	0.013 (0.003, 0.064)	0%	0.027 (0.004, 0.17)	0%	Failed BD Test	Failed BD Test	Failed BD
**16.2.0.0**	0.008 (0.002, 0.038)	0%	0.01 (0.001, 0.068)	0%	0.027 (0.004, 0.17)	0%	Failed BD Test	Failed BD Test	Failed BD Test
**2.0.0.0**	0.019 (0.003, 0.102)	52.77%	0.111 (0.043, 0.257)	60.81%	0.095 (0.019, 0.364)	46.38%	0.138 (0.037, 0.518) P = 0.0022	0.189 (0.035, 1.006) P = 0.11	1.36 (0.347, 5.327) P = 0.91
**2.2.0.0**	0.008 (0.002, 0.038)	0%	0.021 (0.005, 0.079)	0%	0.135 (0.057, 0.286)	0%	0.29 (0.026, 3.2) P = 0.63	0.034 (0.004, 0.306) P = 0.00011	0.123 (0.023, 0.669) P = 0.018
**0.0.0.16**	0.011 (0.002, 0.056)	5.32%	0.022 (0.006, 0.074)	0%	0.039 (0.008, 0.17)	0%	0.289 (0.026, 3.222) P = 0.63	0.218 (0.014, 3.345) P = 0.77	Failed BD Test
**0.16.16.16**	0.006 (0.001, 0.041)	0%	0.013 (0.003, 0.064)	0%	0.027 (0.004, 0.17)	0%	Failed BD Test	Failed BD Test	Failed BD Test
**16.0.0.0**	0.006 (0.001, 0.041)	0%	0.01 (0.001, 0.068)	0%	0.027 (0.004, 0.17)	0%	Failed BD Test	Failed BD Test	Failed BD Test
**16.16.0.0**	0.006 (0.001, 0.041)	0%	0.01 (0.001, 0.068)	0%	0.027 (0.004, 0.17)	0%	Failed BD Test	Failed BD Test	Failed BD Test
**16.18.16.16**	0.006 (0.001, 0.041)	0%	0.01 (0.001, 0.068)	0%	0.027 (0.004, 0.17)	0%	Failed BD Test	Failed BD Test	Failed BD Test
**0.0.16.0**	0.006 (0.001, 0.041)	0%	0.01 (0.001, 0.068)	0%	0.027 (0.004, 0.17)	0%	Failed BD Test	Failed BD Test	Failed BD Test
**0.18.0.0**	0.006 (0.001, 0.041)	0%	0.01 (0.001, 0.068)	0%	0.027 (0.004, 0.17)	0%	Failed BD Test	Failed BD Test	Failed BD Test
**16.16.16.16**	0.006 (0.001, 0.041)	0%	0.01 (0.001, 0.068)	0%	0.027 (0.004, 0.17)	0%	Failed BD Test	Failed BD Test	Failed BD Test
**2.2.16.16**	0.006 (0.001, 0.041)	0%	0.01 (0.001, 0.068)	0%	0.027 (0.004, 0.17)	0%	Failed BD Test	Failed BD Test	Failed BD Test
**2.16.0.0**	0.006 (0.001, 0.041)	0%	0.01 (0.001, 0.068)	0%	0.027 (0.004, 0.17)	0%	Failed BD Test	Failed BD Test	Failed BD Test
**1.0.0.0**	0.006 (0.001, 0.041)	0%	0.01 (0.001, 0.068)	0%	0.027 (0.004, 0.17)	0%	Failed BD Test	Failed BD Test	Failed BD Test
**3.0.0.0**	0.006 (0.001, 0.041)	0%	0.01 (0.001, 0.068)	0%	0.027 (0.004, 0.17)	0%	Failed BD Test	Failed BD Test	Failed BD Test
**18.18.0.0**	0.006 (0.001, 0.041)	0%	0.01 (0.001, 0.068)	0%	0.027 (0.004, 0.17)	0%	Failed BD Test	Failed BD Test	Failed BD Test
**2.18.0.0**	0.006 (0.001, 0.041)	0%	0.01 (0.001, 0.068)	0%	0.027 (0.004, 0.17)	0%	Failed BD Test	Failed BD Test	Failed BD Test
**18.18.16.16**	0.006 (0.001, 0.041)	0%	0.01 (0.001, 0.068)	0%	0.027 (0.004, 0.17)	0%	Failed BD Test	Failed BD Test	Failed BD Test
**0.0.2.0**	0.006 (0.001, 0.041)	0%	0.01 (0.001, 0.068)	0%	0.027 (0.004, 0.17)	0%	Failed BD Test	Failed BD Test	Failed BD Test
**18.2.0.0**	0.006 (0.001, 0.041)	0%	0.01 (0.001, 0.068)	0%	0.027 (0.004, 0.17)	0%	Failed BD Test	Failed BD Test	Failed BD Test

Note: Proportions (95% CI). OR = Odds Ratio

Supplemental Table S4. Cleft Lip and Palate and Laterality with TAC Patterns

	Left CLP	I^2	Right CLP	I^2	Bilateral CLP	I^2	Left vs Right OR	Left vs Bilateral OR	Right vs Bilateral OR
**0.0.0.0**	0.466 (0.415, 0.518)	0%	0.459 (0.387, 0.532)	0%	0.436 (0.358, 0.518)	68.92%	1.031 (0.72, 1.477) P = 0.94	1.065 (0.772, 1.47) P = 0.76	1.038 (0.708, 1.523) P = 0.92
**0.2.0.0**	0.197 (0.159, 0.242)	0%	0.051 (0.018, 0.138)	58.05%	0.083 (0.057, 0.121)	37.84%	4.72 (2.291, 9.723) P = 1e-05	2.226 (1.373, 3.609) P = 0.0014	0.481 (0.22, 1.052) P = 0.091
**0.0.16.16**	0.004 (0.001, 0.021)	0%	0.008 (0.002, 0.04)	0%	0.006 (0.002, 0.02)	0%	Failed BD Test	Failed BD Test	Failed BD Test
**0.1.0.0**	0.004 (0.001, 0.021)	0%	0.006 (0.001, 0.038)	0%	0.003 (0.001, 0.015)	0%	Failed BD Test	Failed BD Test	Failed BD Test
**0.16.0.0**	0.013 (0.003, 0.051)	25.18%	0.008 (0.002, 0.04)	0%	0.024 (0.013, 0.043)	0%	Failed BD Test	0.45 (0.124, 1.629) P = 0.36	0.22 (0.026, 1.867) P = 0.26
**16.2.0.0**	0.003 (0, 0.02)	0%	0.008 (0.002, 0.04)	0%	0.01 (0.004, 0.024)	0%	Failed BD Test	Failed BD Test	Failed BD Test
**2.0.0.0**	0.054 (0.022, 0.127)	73.81%	0.193 (0.142, 0.257)	0%	0.08 (0.059, 0.107)	0%	0.235 (0.13, 0.426) P = 7.6e-07	Failed BD Test	2.831 (1.575, 5.088) P = 0.00057
**2.2.0.0**	0.023 (0.001, 0.268)	86.27%	0.039 (0.003, 0.324)	82.37%	0.117 (0.083, 0.162)	44.8%	0.653 (0.264, 1.612) P = 0.49	Failed BD Test	0.383 (0.177, 0.83) P = 0.019
**0.0.0.16**	0.003 (0, 0.02)	0%	0.006 (0.001, 0.038)	0%	0.007 (0.003, 0.021)	0%	Failed BD Test	Failed BD Test	Failed BD Test
**0.16.16.16**	0.003 (0, 0.02)	0%	0.006 (0.001, 0.038)	0%	0.007 (0.003, 0.02)	0%	Failed BD Test	Failed BD Test	Failed BD Test
**16.0.0.0**	0.011 (0.004, 0.03)	0%	0.006 (0.001, 0.038)	0%	0.014 (0.007, 0.03)	0%	Failed BD Test	0.974 (0.217, 4.366) P = 0.97	Failed BD Test
**16.16.0.0**	0.008 (0.002, 0.026)	0%	0.017 (0.005, 0.051)	0%	0.016 (0.007, 0.033)	0%	0.335 (0.055, 2.029) P = 0.44	Failed BD Test	4.163 (0.436, 39.764) P = 0.4
**16.18.16.16**	0.009 (0.003, 0.026)	0%	0.006 (0.001, 0.038)	0%	0.006 (0.002, 0.02)	0%	Failed BD Test	Failed BD Test	Failed BD Test
**0.0.16.0**	0.004 (0.001, 0.021)	0%	0.006 (0.001, 0.038)	0%	0.006 (0.002, 0.02)	0%	Failed BD Test	Failed BD Test	Failed BD Test
**0.18.0.0**	0.004 (0.001, 0.021)	0%	0.006 (0.001, 0.038)	0%	0.015 (0.007, 0.031)	0%	Failed BD Test	Failed BD Test	Failed BD Test
**16.16.16.16**	0.004 (0.001, 0.021)	0%	0.008 (0.002, 0.04)	0%	0.005 (0.001, 0.018)	0%	Failed BD Test	Failed BD Test	Failed BD Test
**2.2.16.16**	0.006 (0.001, 0.023)	0%	0.008 (0.002, 0.04)	0%	0.006 (0.002, 0.02)	0%	1.017 (0.092, 11.291) P = 0.99	Failed BD Test	Failed BD Test
**2.16.0.0**	0.003 (0, 0.02)	0%	0.017 (0.005, 0.051)	0%	0.005 (0.001, 0.018)	0%	Failed BD Test	Failed BD Test	4.163 (0.436, 39.764) P = 0.4
**1.0.0.0**	0.003 (0, 0.02)	0%	0.019 (0.006, 0.057)	0%	0.007 (0.003, 0.021)	0%	Failed BD Test	Failed BD Test	4.74 (0.477, 47.14) P = 0.35
**3.0.0.0**	0.005 (0.001, 0.023)	0%	0.011 (0.003, 0.044)	0%	0.003 (0.001, 0.015)	0%	0.251 (0.023, 2.802) P = 0.55	Failed BD Test	Failed BD Test
**18.18.0.0**	0.005 (0.001, 0.023)	0%	0.006 (0.001, 0.038)	0%	0.018 (0.01, 0.035)	0%	Failed BD Test	0.191 (0.022, 1.68) P = 0.22	Failed BD Test
**2.18.0.0**	0.003 (0, 0.02)	0%	0.006 (0.001, 0.038)	0%	0.012 (0.005, 0.027)	0%	Failed BD Test	Failed BD Test	Failed BD Test
**18.18.16.16**	0.003 (0, 0.02)	0%	0.009 (0.002, 0.045)	0%	0.013 (0.005, 0.029)	0%	Failed BD Test	Failed BD Test	1.431 (0.095, 21.621) P = 0.79
**0.0.2.0**	0.005 (0.001, 0.023)	0%	0.006 (0.001, 0.038)	0%	0.006 (0.002, 0.02)	0%	Failed BD Test	Failed BD Test	Failed BD Test
**18.2.0.0**	0.003 (0, 0.02)	0%	0.006 (0.001, 0.038)	0%	0.012 (0.005, 0.029)	0%	Failed BD Test	Failed BD Test	Failed BD Test

Note: Proportions (95% CI). OR = Odds Ratio

Supplemental Table S5. TAC Patterns Left Cleft Lip vs. Left Cleft Lip and Palate

	Left CL	I^2	Left CLP	I^2	CLP vs CL OR
**0.0.0.0**	0.727 (0.597, 0.827)	58.3%	0.466 (0.415, 0.518)	0%	0.344 (0.235, 0.504) P = 4.5e-08
**0.2.0.0**	0.101 (0.065, 0.155)	1.86%	0.197 (0.159, 0.242)	0%	2.222 (1.282, 3.853) P = 0.0059
**0.0.16.16**	0.008 (0.002, 0.038)	0%	0.004 (0.001, 0.021)	0%	Failed BD Test
**0.1.0.0**	0.024 (0.003, 0.177)	72.51%	0.004 (0.001, 0.021)	0%	0.142 (0.019, 1.08) P = 0.058
**0.16.0.0**	0.008 (0.002, 0.038)	0%	0.013 (0.003, 0.051)	25.18%	Failed BD Test
**16.2.0.0**	0.008 (0.002, 0.038)	0%	0.003 (0, 0.02)	0%	Failed BD Test
**2.0.0.0**	0.019 (0.003, 0.102)	52.77%	0.054 (0.022, 0.127)	73.81%	2.928 (0.855, 10.034) P = 0.12
**2.2.0.0**	0.008 (0.002, 0.038)	0%	0.023 (0.001, 0.268)	86.27%	5.348 (0.63, 45.412) P = 0.18
**0.0.0.16**	0.011 (0.002, 0.056)	5.32%	0.003 (0, 0.02)	0%	Failed BD Test
**0.16.16.16**	0.006 (0.001, 0.041)	0%	0.003 (0, 0.02)	0%	Failed BD Test
**16.0.0.0**	0.006 (0.001, 0.041)	0%	0.011 (0.004, 0.03)	0%	Failed BD Test
**16.16.0.0**	0.006 (0.001, 0.041)	0%	0.008 (0.002, 0.026)	0%	Failed BD Test
**16.18.16.16**	0.006 (0.001, 0.041)	0%	0.009 (0.003, 0.026)	0%	Failed BD Test
**0.0.16.0**	0.006 (0.001, 0.041)	0%	0.004 (0.001, 0.021)	0%	Failed BD Test
**0.18.0.0**	0.006 (0.001, 0.041)	0%	0.004 (0.001, 0.021)	0%	Failed BD Test
**16.16.16.16**	0.006 (0.001, 0.041)	0%	0.004 (0.001, 0.021)	0%	Failed BD Test
**2.2.16.16**	0.006 (0.001, 0.041)	0%	0.006 (0.001, 0.023)	0%	Failed BD Test
**2.16.0.0**	0.006 (0.001, 0.041)	0%	0.003 (0, 0.02)	0%	Failed BD Test
**1.0.0.0**	0.006 (0.001, 0.041)	0%	0.003 (0, 0.02)	0%	Failed BD Test
**3.0.0.0**	0.006 (0.001, 0.041)	0%	0.005 (0.001, 0.023)	0%	Failed BD Test
**18.18.0.0**	0.006 (0.001, 0.041)	0%	0.005 (0.001, 0.023)	0%	Failed BD Test
**2.18.0.0**	0.006 (0.001, 0.041)	0%	0.003 (0, 0.02)	0%	Failed BD Test
**18.18.16.16**	0.006 (0.001, 0.041)	0%	0.003 (0, 0.02)	0%	Failed BD Test
**0.0.2.0**	0.006 (0.001, 0.041)	0%	0.005 (0.001, 0.023)	0%	Failed BD Test
**18.2.0.0**	0.006 (0.001, 0.041)	0%	0.003 (0, 0.02)	0%	Failed BD Test

Note: Proportions (95% CI). OR = Odds Ratio

Supplemental Table S6. TAC Patterns Right Cleft Lip vs. Cleft Lip and Palate

	Right CL	I^2	Right CLP	I^2	CLP vs CL OR
**0.0.0.0**	0.696 (0.605, 0.774)	0%	0.459 (0.387, 0.532)	0%	0.361 (0.218, 0.597) P = 9.9e-05
**0.2.0.0**	0.033 (0.006, 0.173)	54.89%	0.051 (0.018, 0.138)	58.05%	1.509 (0.395, 5.758) P = 0.77
**0.0.16.16**	0.01 (0.001, 0.068)	0%	0.008 (0.002, 0.04)	0%	Failed BD Test
**0.1.0.0**	0.01 (0.001, 0.068)	0%	0.006 (0.001, 0.038)	0%	Failed BD Test
**0.16.0.0**	0.013 (0.003, 0.064)	0%	0.008 (0.002, 0.04)	0%	Failed BD Test
**16.2.0.0**	0.01 (0.001, 0.068)	0%	0.008 (0.002, 0.04)	0%	Failed BD Test
**2.0.0.0**	0.111 (0.043, 0.257)	60.81%	0.193 (0.142, 0.257)	0%	2.079 (1.011, 4.275) P = 0.06
**2.2.0.0**	0.021 (0.005, 0.079)	0%	0.039 (0.003, 0.324)	82.37%	2.123 (0.428, 10.53) P = 0.56
**0.0.0.16**	0.022 (0.006, 0.074)	0%	0.006 (0.001, 0.038)	0%	Failed BD Test
**0.16.16.16**	0.013 (0.003, 0.064)	0%	0.006 (0.001, 0.038)	0%	Failed BD Test
**16.0.0.0**	0.01 (0.001, 0.068)	0%	0.006 (0.001, 0.038)	0%	Failed BD Test
**16.16.0.0**	0.01 (0.001, 0.068)	0%	0.017 (0.005, 0.051)	0%	Failed BD Test
**16.18.16.16**	0.01 (0.001, 0.068)	0%	0.006 (0.001, 0.038)	0%	Failed BD Test
**0.0.16.0**	0.01 (0.001, 0.068)	0%	0.006 (0.001, 0.038)	0%	Failed BD Test
**0.18.0.0**	0.01 (0.001, 0.068)	0%	0.006 (0.001, 0.038)	0%	Failed BD Test
**16.16.16.16**	0.01 (0.001, 0.068)	0%	0.008 (0.002, 0.04)	0%	Failed BD Test
**2.2.16.16**	0.01 (0.001, 0.068)	0%	0.008 (0.002, 0.04)	0%	Failed BD Test
**2.16.0.0**	0.01 (0.001, 0.068)	0%	0.017 (0.005, 0.051)	0%	Failed BD Test
**1.0.0.0**	0.01 (0.001, 0.068)	0%	0.019 (0.006, 0.057)	0%	Failed BD Test
**3.0.0.0**	0.01 (0.001, 0.068)	0%	0.011 (0.003, 0.044)	0%	Failed BD Test
**18.18.0.0**	0.01 (0.001, 0.068)	0%	0.006 (0.001, 0.038)	0%	Failed BD Test
**2.18.0.0**	0.01 (0.001, 0.068)	0%	0.006 (0.001, 0.038)	0%	Failed BD Test
**18.18.16.16**	0.01 (0.001, 0.068)	0%	0.009 (0.002, 0.045)	0%	Failed BD Test
**0.0.2.0**	0.01 (0.001, 0.068)	0%	0.006 (0.001, 0.038)	0%	Failed BD Test
**18.2.0.0**	0.01 (0.001, 0.068)	0%	0.006 (0.001, 0.038)	0%	Failed BD Test

Note: Proportions (95% CI). OR = Odds Ratio
